# A First Case of Compulsive Amnestic Sleep-Related Shopping Induced by Pramipexole Therapy of Restless Legs Syndrome

**DOI:** 10.1055/s-0046-1829640

**Published:** 2026-10-20

**Authors:** Michael Todd, Carlos H. Schenck, Erin Golden, Ranji Varghese

**Affiliations:** 1 Minnesota Regional Sleep Disorders Center, Minneapolis, MN, United States; 2 Department of Psychiatry, Hennepin County Medical Center and University of Minnesota Medical School, Minneapolis, MN, United States; 3 Department of Neurology, Hennepin County Medical Center and University of Minnesota Medical School, Minneapolis, MN, United States

**Keywords:** RLS, compulsive shopping, pramipexole, amnestic sleep related

## Abstract

Dopamine receptor agonists (DAs) such as pramipexole/ropinirole are recognized treatments for restless legs syndrome (RLS) and Parkinson's disease (PD). It is well established that these medications can cause impulse control disorders (ICDs) during wakefulness, side effects with serious and potentially life-altering consequences. Impulse control disorders are recurrent difficult-to-resist behaviors that can be harmful to the patient or others around them. Common examples include pathologic gambling and compulsive sexual behaviors, eating, and shopping. We present the case of a patient with RLS who developed pathologic nocturnal sleep-related shopping, without recalling the events, which resolved after discontinuing pramipexole. This case illustrates the devastating consequences that unrecognized medically-induced ICDs can have on patients' lives and the importance of counseling patients about ICDs during wake and sleep prior to starting DA therapy.

## Case Report

A 58-year-old woman presented with a 28-year history of RLS, for which she was initially prescribed pramipexole. She denied prior history of parasomnias or compulsive behaviors. After starting pramipexole, she reported episodes of pathologic nocturnal shopping, without recollection of the events. She suffered from these events for nearly two decades, causing strain on her mental health, finances, and marriage. The patient denied symptoms concerning sleep-disordered breathing, psychiatric disorders, or seizures. She did not snore and had low probability of sleep-disordered breathing, being given a Snoring, Tiredness, Observed apnea, Pressure, BMI, Age, Neck circumference, Gender (STOP-BANG) score of 1. Additionally, given the lack of stereotypy between episodes, focal seizures were also considered unlikely. One of the authors who eventually managed her ongoing care (RV) is a psychiatrist and sleep specialist who did not detect any symptoms suggestive of a dissociative disorder or other psychiatric comorbidities that could interfere with therapeutic decisions in the long term. Also, there was no clinical indication to perform video-polysomnography.

The first episode occurred when the patient was a graduate student and had fallen asleep on the couch watching TV late one night. The next morning, she had no recollection of what she had watched, nor any memory of placing orders. However, in the next few days, she started receiving packages of different kitchen knives. She discovered that she must have fallen asleep during an infomercial selling kitchen knives. As this event occurred prior to the use of cell phones, she deduced that she must have used a landline to place a phone call and give someone her credit card number and shipping information. However, she had no recollection of having done so.

As time went on, these events continued to occur. For example, she would awake each morning to discover orders placed online at various popular shopping websites. Unnecessary items such as T-shirts, an expensive pool table, and “inappropriate” (her words) clothing would later arrive at her home daily. These items were also ordered in exceptionally high quantities, such as 60 kitchen knives or multiple sets of parts for a vacuum cleaner she never owned. She described suffering daily dread of driving home from work wondering what packages she would find waiting for her. The time-stamped purchases took place, on average, 1 hour after sleep onset, and she continued to suffer from complete event amnesia.

During the day, she took protective and compensatory measures to thwart her nocturnal shopping. One strategy involved removing credit cards from common retail websites and returning the items that arrived. However, during the events, she navigated around the safeguards placed. Her “night self” deleted order confirmation emails, purchased personalized items that could not be returned, and ordered from obscure online retailers she could not trace during the day.

She reported feeling ashamed of what was happening but was too embarrassed to discuss it with her doctor. Eventually, the nocturnal shopping caused strain on her marriage and drove her into debt. After hearing stories of impulsive behavior from other patients taking pramipexole, she disclosed her symptoms to her doctor. A physician (not at our sleep center) switched her medication from pramipexole to ropinirole, which stopped the nocturnal impulsive shopping behaviors. Ropinirole was also effective in controlling her RLS, without augmentation. The nocturnal shopping behavior has not re-occurred since discontinuing pramipexole. It is also notable that despite taking pramipexole for approximately three decades, she never developed augmentation as a side effect, nor was there any augmentation with the more temporally limited use of ropinirole.

## Discussion

Dopamine agonist-induced cases of ICD, including impulsive shopping, have been well documented in wakefulness in patients with RLS and PD. 1 To the best of our knowledge, this case herein described is the first of amnestic nocturnal sleep-related shopping secondary to a DA.

Dopamine agonists such as cabergoline, bromocriptine, rotigotine, pramipexole and ropinirole have each been implicated in causing ICD in wakefulness. Within this group, pramipexole followed by ropinirole had the strongest association with ICD. 1 This has been shown to be consistent for patients with PD or RLS taking these medications. 2 3

Pramipexole and ropinirole bind preferentially to pre- and postsynaptic D2 and D3 dopamine receptors; however, they both share a greater affinity for D3 receptor. The other DAs, bromocriptine and cabergoline, have higher affinity for D2 receptor, while rotigotine acts on receptors D1 to D3. 4 5 6

For the patient in the case currently reported, ICD symptoms resolved when switching from pramipexole to ropinirole. Despite their similarities, we hypothesize that there are still pharmacological differences between these two drugs to account for her improvement. One prospective case control study of RLS patients who developed ICD while taking DAs showed a significantly higher rate of ICD in patients taking pramipexole compared with ropinirole. 3 Another retrospective study of 710 patients with ICD taking DA showed the strongest association with pramipexole. 1

Compared with the D2 receptor, the D3 receptor is heavily expressed in the limbic system, giving it strong influence over emotional processing, reward, and decision making. 7 8 Although both pramipexole and ropinirole have greater affinity for D3 than D2 receptors, this affinity is greater for pramipexole. 9 10 Table 1 compares the pharmacology of ropinirole and pramipexole.

**Table 1:** Pharmacology of ropinirole versus pramipexole. 9 10 11 12 13 14

Characteristic	Ropinirole	Pramipexole
Receptor affinity - D3	K _i_ = 69 nM	K _i_ = 0.5 nM
Receptor affinity - D2	K _i_ = 1,380 nM	K _i_ = 3.9 nM
Bioavailability	∼ 50%	> 90%
Metabolism	Inactivated in liver by CYP 1A2; no pharmacologically active metabolites	Negligible metabolism
Excretion	<10% excreted unchanged in urine	> 90% excreted unchanged in urine
Pharmacokinetics	Linear	Linear
Half life	∼ 6 hours	∼ 8 hours

**Note** : Lower inhibition constant (K _i_ ) value implies stronger binding affinity.

A cross-sectional study of patients with RLS taking DAs showed predisposing factors to ICD development, including higher dose, younger age, female sex, experimental drug use, and family history of gambling. 11 It is likely that the combination of young age, sex, and use of pramipexole were each factors contributing to the patient in this case developing ICD. Switching from pramipexole, a medication with a very high affinity for the D3 receptor, to ropinirole, a medication with a relatively lower D3 affinity, is likely the reason why ICD resolved for the patient presented in this case. It should be noted that the decision to switch from one DA to another was made by a non-sleep medicine-trained physician. A switch to an α-2-delta ligand calcium channel blocker (gabapentin, pregabalin) would have been more prudent. Nevertheless, this debatable clinical decision turned out to be quite informative, both clinically and scientifically, and beneficial for the patient.

## Conclusion

The case herein reported is the first of pramipexole-induced amnestic nocturnal sleep-related ICD (specifically involving shopping by phone or online), with severe negative impacts. The symptomatology can be so unusual and embarrassing that patients might be reluctant to discuss this relentless problem with their prescribing doctor. When prescribing DAs, providers should counsel patients on the symptoms of ICD (including shopping), with manifestations emerging during wakefulness and sleep.
